# Prognostic value of cardiac magnetic resonance in patients with aortic stenosis: A systematic review and meta-analysis

**DOI:** 10.1371/journal.pone.0263378

**Published:** 2022-02-03

**Authors:** Chuan Zhang, Jie Liu, Shu Qin

**Affiliations:** 1 Department of Cardiology, The First Affiliated Hospital of Chongqing Medical University, Yuzhong District, Chongqing, China; 2 Chongqing Municipal Health Supervision Bureau, Chongqing, China; Universitatsklinikum Wurzburg, GERMANY

## Abstract

**Background:**

The timing of surgery for aortic stenosis (AS) is imperfect, and the management of moderate AS and asymptomatic severe AS is still challenging. Myocardial fibrosis (MF) is the main pathological basis of cardiac decompensation in patients with AS and can be detected by cardiovascular magnetic resonance (CMR). The aim of this study was to evaluate the prognostic value of MF measured by CMR in patients with AS, which can provide a reference for the timing of aortic valve replacement (AVR).

**Methods:**

We searched Medline, Embase, and Web of Science to include all studies that investigated the prognostic value of CMR in patients with AS. The search deadline is March 31, 2021. The pooled relative risk (RR) or hazard ratio (HR) and 95% confidence intervals (CI) of the biomarkers including late gadolinium enhancement (LGE), Native T1 or extracellular volume (ECV) were calculated to evaluate the prognostic value.

**Results:**

13 studies and 2,430 patients with AS were included in this study, the mean or medium follow-up duration for each study was ranged from 6 to 67.2 months. Meta-analysis showed the presence of LGE was associated with an increased risk for all-cause mortality (pooled RR: 2.14, 95% CI: 1.67–2.74, P < 0.001), cardiac mortality (pooled RR: 3.50, 95% CI: 2.32–5.30, P < 0.001), and major adverse cardiovascular events (MACEs) (pooled RR: 1.649, 95% CI: 1.23–2.22, P = 0.001). Native T1 was significantly associated with MACEs (pooled RR: 2.23, 95% CI: 1.00–4.95; P = 0.049), and higher ECV was associated with a higher risk of cardiovascular events (pooled HR: 1.69, 95% CI: 1.11–2.58; P = 0.014).

**Conclusion:**

The use of CMR to detect MF has a good prognostic value in patients with AS. LGE, Native T1 and ECV measured by CMR can contribute to risk stratification of AS, thereby helping to optimize the timing of AVR.

## Introduction

Aortic stenosis (AS) is the most common valvular diseases, and the prevalence in elderly is about 6%– 12.4% [1, 2]. Due to the aging of the population, its prevalence is gradually increasing. Currently, the main treatment that can definitely improve the prognosis is aortic valve replacement (AVR). The appearance of symptoms is associated with an increased morbidity and mortality [3–5]. The recent guidelines recommended AVR should be early performed in all symptomatic patients with severe AS because of their dismal spontaneous prognosis [6]. But the early stage of AS is usually long and asymptomatic, once patients with AS develop clinical symptoms, the condition usually develops rapidly and is difficult to reverse. Management of asymptomatic AS remains controversial, AVR is indicated in patients with depressed left ventricular function (left ventricular ejection fraction (LVEF) < 50%) not due to other causes or in patients with symptoms during exercise testing [7, 8]. However, since the elderly often develop other cardiovascular diseases at the same time, it is difficult to identify whether the symptoms are caused by AS. In addition, irreversible myocardial damage may occur before symptoms appear or LVEF decreases. It should be noted that moderate AS still lacks effective management. On the other hand, patients with AS are generally older and the risk of AVR is higher, so it is not suitable for all asymptomatic patients. Therefore, it is very important to find new effective markers to identify the degree of myocardial damage in patients with AS at an early stage.

Pathological basis of AS includes both valvular stenosis and myocardial remodeling. Moderate to severe AS results in left ventricular pressure overload, which leads to adaptive remodeling of the heart to maintain left ventricular wall stress and cardiac output. Over time, Supply-demand ischemia results in cardiomyocyte death and myocardial fibrosis (MF), which ultimately leads to left ventricular dysfunction and symptoms [9, 10]. MF is the key pathological basis leading to left ventricular decompensation and is related to the poor long-term prognosis of AS [11–13]. Myocardial biopsy is currently the gold standard for detecting MF, but it is an invasive procedure with the risk of complications, so it cannot be widely used clinically. Cardiac magnetic resonance (CMR) provides the best way to non-invasively detect MF [14]. The main biomarkers for CMR assessment MF include late gadolinium enhancement (LGE), Native T1 and extracellular volume (ECV). Among them, Native T1 and ECV are promising imaging biomarkers in detecting diffuse MF in AS, and have become a research hotspot in predicting the prognosis of AS in recent years.

The purpose of this study is to evaluate whether LGE, Native T1 or ECV measured by CMR is related to cardiovascular adverse events in AS, so as to provide a reference for the timing of AVR.

## Methods

### Search strategy

In the current systematic review and meta-analysis, two independent reviewers (J Liu and C. Zhang) systematically searched Medline (via PubMed), Embase, web of science until March 31, 2021. The main search terms included “aortic stenosis” AND “CMR, cardiovascular magnetic resonance” AND “myocardial or cardiac fibrosis” AND “prognosis OR outcome OR mortality OR cardiovascular events OR heart failure”. The search was limited to studies in humans, original papers, prospective observational studies and with available full-text. We also reviewed the references cited in the search article for additional articles.

### Study selection

We did our best to include all studies on MF detected by CMR. End points should include one of the following outcomes: all-cause mortality, cardiac mortality, and major adverse cardiovascular events (MACEs, including all cause death, cardiac death, non-fatal myocardial infarction, sustained ventricular arrhythmias, third-degree atrioventricular block and hospitalization for heart failure). Studies in vitro or animal models were excluded. The eligibility of studies for inclusion in this meta-analysis were independently assessed by two reviewers (J. Liu and C. Zhang) using standardized protocols. All titles and abstracts were independently reviewed by these two reviewers to assess their eligibility and obtain the full text of articles that were potentially eligible for inclusion. The two reviewers would discuss the differences of included articles to achieve agreement or seek the opinion of a third person (S Qin) to decide. Eligible studies were based on the following: patients meet the diagnostic criteria for moderate or severe AS, which is defined by echocardiography as transaortic peak velocity greater than 3.0 m/s or transaortic mean pressure gradient greater than 20 mmHg or aortic valve stenosis less than 1.5cm^2^ [15]; detection of MF by CMR to predict the prognosis or outcomes in patients with AS, at least one of LGE, Native T1 or ECV should be included as a biomarker for measuring MF; the study endpoint included at least one of the three results of the AS described above. Studies with less than 10 subjects or less than 3 months of follow-up were excluded.

### Data extraction

The data was extracted and processed by the two researchers independently (J. Liu and C. Zhang) in the standardized data collection forms. Items included date, the first author, year of publication, publication type, type of study, number of involved centers (single or multi-centre), objective, sample size, inclusion and exclusion criteria, patient baseline characteristics, AS grade, imaging biomarker, follow-up period, and research endpoint such as all-cause mortality, cardiac mortality, and MACEs. If the endpoint is not clearly reported, we will contact the research investigator directly to obtain the raw data. The data extracted by the two researchers were compared and summarized, and the differences between the two researchers were resolved through discussion.

### Quality assessment

All the included studies were prospective cohort studies. Two investigators reviewed the studies and assessed the quality and risk of bias in accordance with the Newcastle-Ottawa Quality Assessment Scale (NOS) [16]. The tool includes 8 items in three categories: crowd selection (4 items), comparability (1 item), outcome evaluation (3 items). For each numbered item in the crowd selection and outcome evaluation, a study can earn up to one star. A maximum of two stars can be given for comparability. The study obtained seven or eight stars was considered high quality and nine stars represented the best quality. The disagreement between the two investigators was resolved through discussion.

### Data analysis

The current systematic review and meta-analysis were performed in accordance with guidelines of the MOOSE (Meta-analysis of Observational Studies in Epidemiology) and PRISMA (Preferred Reporting Items for Systematic Reviews and Meta-Analyses) [17, 18]. Dichotomous variables were reported as proportions; continuous variables were reported as mean ± SD (Standard deviation). The effect of each study was expressed by relative risk (RR), hazard ratio (HR) and the accompanying 95% confidence intervals (CI). In articles where the original number of clinical events cannot be obtained, we chose HR or OR calculated through multivariate analysis or adjusted value. Chi-square test and I^2^ were used to measure the Statistical heterogeneity between studies. I^2^ statistic > 50% or P < 0.05 indicated the presence of heterogeneity. Publication bias was assessed with funnel plots, Egger’s test [19]. A publication bias would be detected if the funnel plot was asymmetric and Egger’s test had a p < 0.05. Statistical analysis was performed by using STATA 15.0 (Stata Corporation, College Station, Texas). We selected statistical models based on the heterogeneity of the I^2^ assessment. If there was no significant heterogeneity between the included studies, the combined RR, HR, and 95% CI were calculated by the Mantel-Haenszel method using a fixed effect model. If there is significant heterogeneity between the studies, the D-L (DerSimonian-Laird) method using the random effect model is chosen. Statistical significance for hypothesis testing was set at 0.05 (two-tailed), the hypothesis test value of the pooled effects < 0.05 was considered statistically significant.

## Results

### Results of the literature search

**Fig 1:**
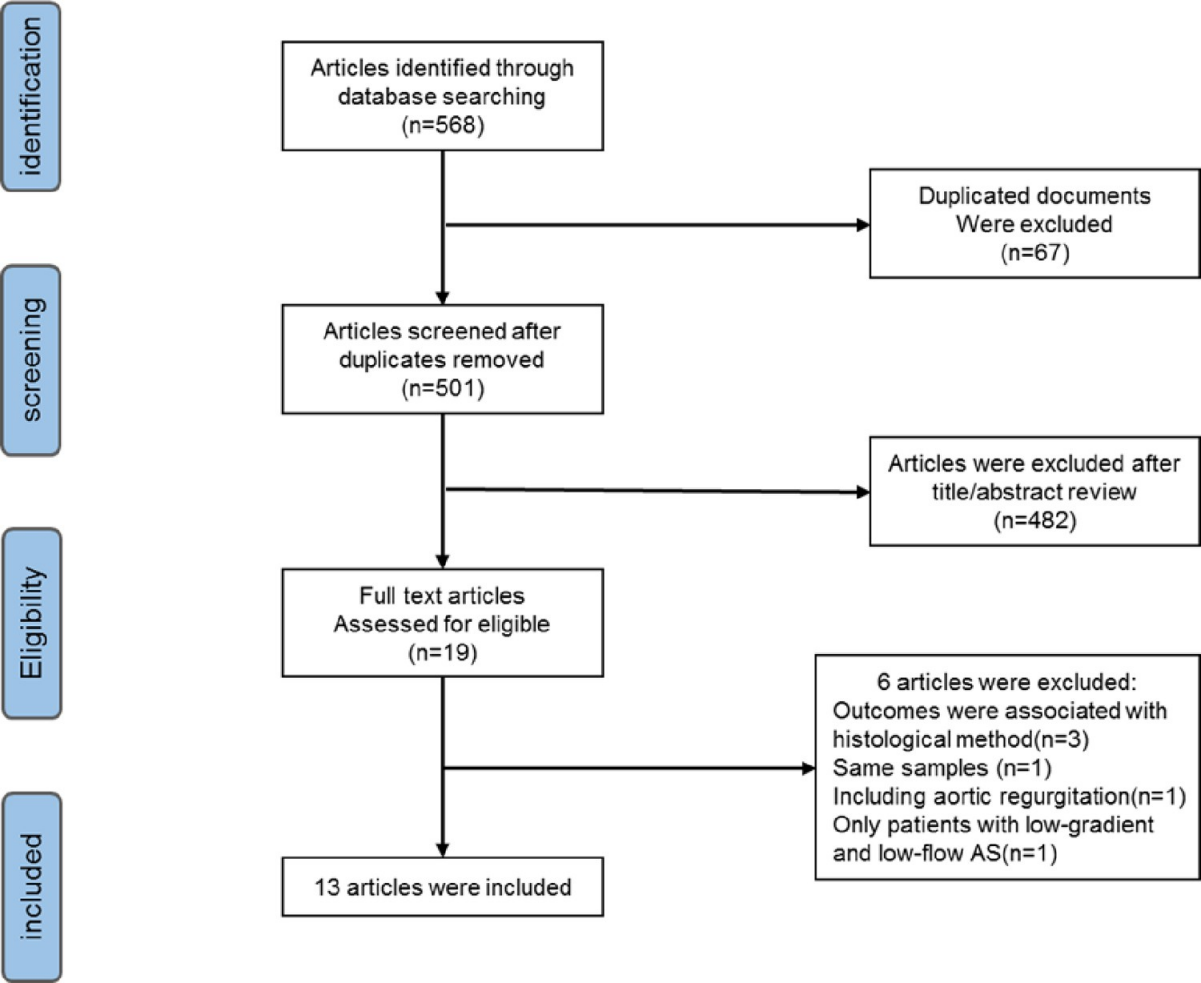
Flow diagram of the literature search process.

The final search was on March 31, 2021. Our literature search identified 568 relevant abstracts. By screening titles and abstracts, duplicate articles, unrelated topics, in vitro and animal research articles, and articles without full text were excluded. We carefully reviewed the remaining 19 full-text articles. Finally, 3 of these studies were excluded because the clinical outcomes were primarily associated with myocardial fibrosis detected by histological method. One study was excluded because only a subset of patients with low-gradient and low-flow AS were evaluated [20]. We excluded a study that included 54 patients because 6 of 28 patients with severe AS also exhibited moderate aortic regurgitation [21]. Two studies have same sample, and the study was selected for it contains more detailed data [22, 23]. The left 13 studies for detailed analysis. A flow diagram detailing the search can be found in Fig 1.

### Study characteristics

Total number of 2,430 patients with AS were included in this review, and the median number of patients per study was 187 patients (range 43 to 674). The mean or medium follow-up duration for each study was ranged from 6 to 67.2 months. The demographics and characteristics of the patients with AS was presented in Table 1.

**Table 1:** Patient demographics and characteristics.

First author	Year of publication	N	Age (years)	Male n (%)	Flow up (months)	AVA (cm^2^) or AVAI (cm^2^/m^2^)	P Max (mmHg)	P Mean (mmHg)	LVEF (%)
Hyun-Jung Lee [24]	2021	191	68.4 ± 8.8	96 (50.3%)	67.2	0.78 ± 0.23	NR	54.0 ± 21.5	62.6±13.0
Russell [25]	2020	440	70 ± 10	259 (59%)	45.6	0.73 ± 0.25	82.0± 29.3	49.7 ± 18.7	66 ± 12
Hwang [28]	2019	43	65.9± 8.1	24 (55.8%)	38.8	AVAI: 0.45 ± 0.13	NR	50.4 ± 7.3	64.9±11.2
Agoston-Colde [31]	2019	52	66 ± 7.5	29 (55.7)	12.7	0.52 ± 0.08	82.1 ± 17.9	52.9 ± 14.7	58.4 (9.7)
Musa [26]	2018	674	74.6 ± 14.4	425 (63.1)	43.2	0.70 ± 0.31	78.0 ± 30.0	46 ± 18	61.0 ± 16.7
Chin [29]	2017	166	69 ± 6	115 (69)	34.8	1.0 ± 0.4	NR	35 ± 19	65 (62–68)
Heesun Lee [30]	2017	127	68.8 ± 9.2	63 (49.6)	27.9	0.82 ± 0.25	NR	48 ± 19.3	60.1 ± 9.7
Rajesh [32]	2017	109	57.3 ± 12.5	63 (57.8)	13	NR	73.5 ± 23.0	44.7 ± 13.6	56.5 ± 12.4
Singh [27]	2017	174	66.2 ± 13.34	133 (76)	12.3	AVAI:0.57 ± 0.14	NR	35.4 ± 12.5	56.7 ± 3.7
Nadjiri [33]	2016	94	80 ± 5	55 (59)	6	NR	NR	NR	56 ± 16
Barone-Rochette [34]	2014	154	74 ± 9	96 (62)	34.8	0.71 ± 0.17	79 ± 25	49 ± 17	60 ± 15
Quarto [35]	2012	63	72.4 ± 11	47 (75)	24	0.89 ± 0.27	NR	NR	57.7 ± 17.6
Dweck [23]	2011	143	68 ± 14	97 (68)	24	0.99 ± 0.31	69.7 ± 23.4	NR	57.8 ± 20.2

*N = sample size; AS = aortic stenosis; AVA = aortic valve area; AVAI = index aortic valve area; P Max = maximal transvalvular pressure gradient; P Mean = mean transvalvular pressure gradient; NR = not reported; LVEF = left ventricular ejection fraction.

9 out of 13 studies are single-center prospective studies, and 4 are multi-center prospective studies [24–27]. In 4 studies, patients with AS underwent CMR using a 3.0T scanner [27–30]. In 2 studies, patients underwent CMR at 1.5 or 3.0 T scanner [24–26]. The remaining 6 studies, patients underwent CMR at 1.5T scanner. The baseline characteristics of the included studies was presented in Table 2.

**Table 2:** Baseline characteristics of the studies included in the meta-analysis.

First author	Year of publication	Country	Study design	Imaging biomarker	Outcomes	Field strength	Quality assessment score
Hyun-Jung Lee [24]	2020	Republic of Korea	Prospective, Multi-centre	ECV	MACEs	1.5T/3.0T	8
Everett [25]	2020	Europe	Prospective, Multi-centre (10 centers)	LGE	ACM, CM	1.5T/3.0T	9
		North America		ECV	ACM, CM		
		Asia		Native T1	ACM, CM		
Hwang [28]	2019	Republic of Korea	Prospective, Single-centre	Native T1	MACEs	3.0T	7
Agoston-Colde [31]	2019	Romania	Prospective, Single-centre	LGE	MACEs	1.5T	8
Musa [26]	2018	United Kingdom	Prospective, Multi-centre	LGE	ACM, CM	1.5T/3.0T	9
Chin [29]	2017	United Kingdom	Prospective, Single-centre	LGE	ACM, CM	3.0T	9
				ECV	ACM, CM		
Heesun Lee [30]	2017	Republic of Korea	Prospective, Single-centre	LGE	MACEs	3.0T	8
				Native T1	MACEs		
Rajesh [32]	2017	Indian	Prospective, Single-centre	LGE	MACEs, ACM	1.5T	7
Singh [27]	2017	United Kingdom	Prospective, Multi-centre	LGE	MACEs	3.0T	8
				ECV	MACEs		
				Native T1	MACEs		
Nadjiri [33]	2016	Germany	Prospective, Single-centre	ECV	ACM, HF	1.5T	
				Native T1	MACEs		
Barone-Rochette [34]	2014	Belgium	Prospective, Single-centre	LGE	ACM, CM	1.5T	9
Quarto [35]	2012	United Kingdom	Prospective, Single-centre	LGE	MACEs, ACM	1.5T	7
Dweck [23]	2011	United Kingdom	Prospective, Single-centre	LGE	ACM, CM	1.5T	8

*LGE = late gadolinium enhancement, ECV = extracellular volume, MACEs = major adverse cardiovascular events, CM = cardiac mortality, HF = heart failure.

### Study quality

All studies recorded were of low risk. Four studies received 9 stars in NOS, and the remaining studies received 7 to 8 stars. In some studies, the comparability of cohorts based on design or analysis was not well evaluated and only one star was obtained. A total of 7 studies reported the use of blinded analysis and evaluation by at least two analysts. The follow-up duration for one study was only 6 months and the scores were also lost (S1 Table).

### LGE and cardiovascular outcomes

**Fig 2:**
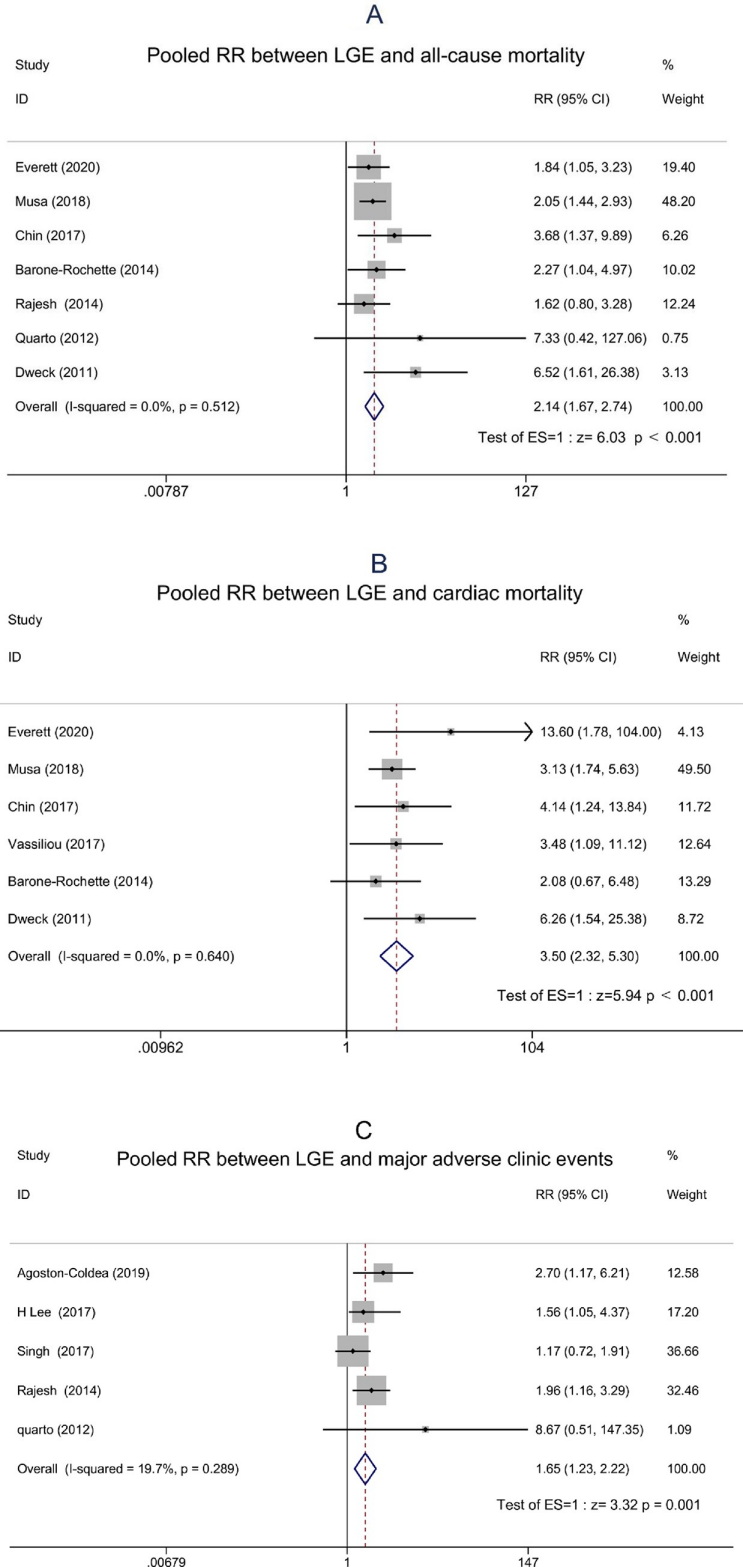
Forest plots showing the pooled relative risks between LGE and outcomes in patients with AS. (A) The pooled RR between LGE and all-cause mortality. (B) The pooled RR between LGE and cardiac mortality. (C) The pooled RR between LGE and MACEs. *RR relative risk, LGE late gadolinium enhancement, MACEs major adverse cardiovascular events.

Standard meta-analysis of outcomes in patients with AS were performed if sufficient data was available (at least 2 studies). Patients in 10 studies underwent LGE [23, 25–27, 29–32, 34, 35]. There was a significant association between LGE and all-cause mortality, cardiac mortality, and MACEs based on the combined RR in a fixed effect model (RR: 2.14, 95% CI: 1.67–2.74, P < 0.001; RR: 3.50, 95% CI: 2.32–5.30, P < 0.001; RR: 1.65, 95% CI: 1.23–2.22, P = 0.001, respectively; Fig 2A–2C). Mild heterogeneity was detected (I^2^ = 0.0%, I^2^ = 0.0%, I^2^ = 19.7%, respectively).

### Native T1 mapping and cardiovascular outcomes

**Fig 3:**
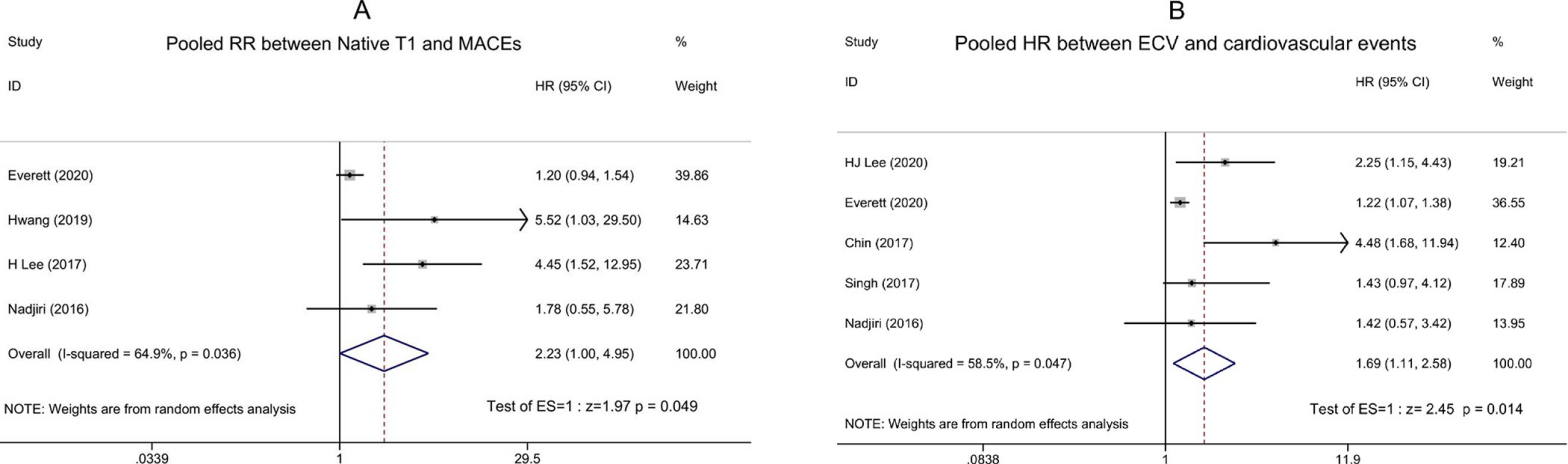
Forest plots showing the pooled RR or HR between Native T1 or ECV and cardiovascular events in patient with AS. (A) The pooled HR between Native T1 and MACEs; (B) The pooled HR between ECV and cardiovascular events. * RR relative risk, HR hazard ratio, MACEs major adverse cardiovascular events, ECV extracellular volume.

4 of the 13 studies reported the prognostic value of the Native T1 for MACEs [23, 26, 28, 31]. Moderate heterogeneity was detected (I^2^ = 64.9%, P = 0.036). Patients with higher T1 values had a significantly higher risk of MACEs during follow-up than those with lower T1 values (RR: 2.23, 95% CI: 1.00–4.95; P = 0.049, Fig 3A) in the random effect model.

### ECV and cardiovascular outcomes

5 of the 13 studies reported ECV in patients with AS and were included in the meta-analysis [24, 25, 27, 29, 33]. We investigated the association between ECV and cardiovascular events. Moderate heterogeneity was detected (I^2^ = 58.5%, P = 0.047). In the random effect model, the combined HR was 1.69 (95% CI: 1.11–2.58; P = 0.014, Fig 3B). This suggested that a higher ECV was associated with a higher risk of cardiovascular events.

### Publication bias

The funnel plots showed that all outcomes for LGE, Native T1 and ECV were equally distributed around the overall estimate. Moreover, the Egger’s test showed no significant publication bias (S1 Fig).

## Discussion

In this study, we performed a meta-analysis of MF by CMR and prognosis of patients with AS. The results showed that MF measured by CMR was a good predictor of AS prognosis. LGE, Native T1 and ECV can contribute to track myocardial health in patients with AS, thereby helping to optimize the timing of AVR.

Due to the aging population, AS is the most common heart valve disease [36]. Currently, the most effective treatment for AS is still AVR which is mainly recommended in symptomatic patients with severe, high-gradient AS [37]. However, these indicators are sometimes poorly correlated with the condition of the disease and depend on stroke volume. Patients with AS are usually asymptomatic in the early stage and have a good prognosis, but when patients develop symptoms are associated with substantial morbidity and mortality [3–5]. This indicates that for some asymptomatic patients, irreversible heart damage may have occurred during the follow-up of clinical symptoms. The degree of heart damage cannot be assessed based on the degree of AS alone, so it is necessary to develop new biomarkers to detect early signs of LV decompensation. MF is characterized by an increase in myocardial interstitial collagen volume [38] under various pathological factors (such as AS), which is a key pathological basis for heart failure and arrhythmia [39]. The study by Milano et al. showed that myocardial fibrosis is related to adverse cardiovascular events in AS [40]. Therefore, MF may be a good marker for assessing the risk of AS.

CMR provides detailed tissue characterization, can identify MF non-invasively, and is closely related to MF measured by histology [41]. In recent years, it has been widely used to predict the prognosis of AS.

LGE is the most widely used CMR method for detecting MF. It can detect replacement fibrosis of the myocardium, which is generally considered irreversible after AVR [42]. Many previous studies have shown that LGE is an independent predictor of adverse events in patients with AS [25, 26]. The present meta-analysis demonstrated significant association between LGE and cardiovascular events. Presence of LGE was associated with an increased risk for all-cause mortality, cardiac mortality, and MACEs.

There are two different modes of MF: reactive interstitial fibrosis (diffuse fibrosis) and replacement fibrosis. Diffuse fibrosis is usually an early stage of replacement fibrosis and is considered reversible [43, 44]. In contrast, replacement fibrosis usually occurs later than diffuse fibrosis and is generally considered irreversible [45]. Treibel et al. [46] reported that the degree of replacement fibrosis after AVR was not reduced, but diffuse fibrosis and cardiomyocyte hypertrophy regressed. Therefore, AVR may be more beneficial after diffuse fibrosis has occurred than after alternative fibrosis has occurred.

LGE relies on the contrast between healthy and diseased myocardium, so it is difficult for LGE to quantify the level of diffuse MF. T1 mapping based on T1 relaxation time which is the magnetic properties of the tissue and its surrounding environment, so it can be used to evaluate diffuse MF [47, 48]. This meta-analysis showed that Native T1 was significant associated with the MACEs (Fig 3A). The study from Lee et al. [30] showed Native T1 was higher in patients with AS than control subjects (1,232 ± 53ms vs. 1,185 ± 37ms; p = 0.008), and the Native T1 was a predictor of poor prognosis (adjusted HR for every 20ms increase of Native T1: 1.28; 95% CI: 1.10–1.46, p = 0.003). However, Native T1 depends on many factors, such as the age and gender of the patient, acquisition sequence, scanner field strength, and post-processing. There was also a large overlap in Native T1 values between patients with AS and healthy controls [49]. Therefore, there is no uniform diagnostic threshold in evaluating the level of MF in patients with AS and further research is needed to improve uniform diagnostic criteria.

ECV is derived from T1 mapping and can also be used to detect diffuse MF. Combining Native T1, contrast-enhanced T1 and hematocrit, ECV can be calculated according to a formula [50–52]. ECV represents the percentage of extracellular matrix in the entire myocardium, therefore it has better repeatability than Native T1 and is comparable in different studies. Everett [25] enrolled 440 patients with severe AS scheduled for AVR, they found increased ECV was a good indicator of left ventricular decompensation and a powerful independent predictor of mortality. This meta-analysis showed that ECV was a good predictor of the prognosis of AS patients (Fig 3B).

As mentioned above, the role of CMR in predicting the prognosis of patients with AS has been demonstrated. CMR can detect diffuse and replacement fibrosis of the myocardium non-invasively, this is helpful for risk stratification of patients with AS and provides a reference for obtaining more precise treatments such as AVR. According to existing clinical guidelines, patients with symptomatic severe AS should receive AVR. However, there are still challenges in the timing of AVR for patients with moderate AS or asymptomatic severe AS. For these patients, while waiting for symptoms to appear, ventricular remodeling may progress, CMR can screen high-risk patients early to optimize the timing of AVR, and improve patient prognosis. A study by Hwang et al. [28] showed that 18 out of 30 patients with elevated native T1 returned to normal after receiving AVR. The Native T1 significantly decreased 1 year after AVR (pre-AVR, 1233.8 ± 49.7ms; post-AVR, 1189.1 ± 58.4ms; P< 0.001), which was associated with left ventricular mass regression and systolic function improvement.

This meta-analysis had some limitations. There were fewer articles about Native T1 and ECV for predicting prognosis of AS, which may lead to bias in meta-analysis results. Even so, the current research provided new ideas for the management of patients with AS patients including moderate AS or asymptomatic severe AS. A clinical study (EVOLVED) on LGE to optimize the timing of AVR is ongoing [53]. In the future, more large-scale prospective studies are needed to clarify the specific method of using CMR to optimize the timing of AVR before irreversible heart damage occurs.

## Conclusion

The timing of surgery for AS is imperfect, and the management of moderate AS and asymptomatic severe AS is still challenging. MF plays a key role in the pathophysiology of AS. LGE is a mature CMR technique for detecting replacement MF and has been demonstrated as a good predictor of prognosis in patients with AS in many studies. Native T1 and ECV are promising CMR technique in detecting diffuse MF in AS, which can detect myocardial structural abnormalities earlier. MF assessed by CMR is a good indicator for predicting the prognosis of AS, and can be used to track myocardial health for risk stratification. This will help optimize the timing of AVR for AS patients, especially asymptomatic patients with severe AS or moderate AS.

## Supporting information

S1 Checklist PRISMA 2020 checklist. (DOCX)

S2 Checklist PRISMA 2020 for abstracts checklist. (DOCX)

S1 Fig Egger’s publication bias plots. (A) Egger’s test for LGE and all-cause mortality. (B) Egger’s test for LGE and cardiac mortality. (C) Egger’s test for LGE and MACEs. (D) Egger’s test for ECV and cardiovascular events. (E) Egger’s test for native T1 and MACEs.
(DOCX)

S1 Table Risk of bias within studies. (DOCX)

S2 Table Search strategy (via PubMed). (DOCX)

## Abbreviations

CMR: Cardiac magnetic resonance
AS: Aortic stenosis
LGE: Late gadolinium enhancement
ECV: Extracellular volume
RR: Relative risk
HR: Hazard ratio
CI: Confidence intervals
AVR: Aortic valve replacement
LVEF: Left ventricular ejection fraction
MACEs: major adverse cardiovascular events
MF: Myocardial fibrosis
MOOSE: Meta-analysis of Observational Studies in Epidemiology
PRISMA: Preferred Reporting Items for Systematic Reviews and Meta-Analyses
SD: Standard deviation
