# Supplementary material for: Prognostic value of cardiac magnetic resonance in patients with aortic stenosis: A systematic review and meta-analysis
Source: PLoS One. 2022 Feb 3;17(2):e0263378. doi: 10.1371/journal.pone.0263378 (PMC8812989; doi:10.1371/journal.pone.0263378)
Supplement: S1 Fig — (A) Egger’s test for LGE and all-cause mortality. (B) Egger’s test for LGE and cardiac mortality. (C) Egger’s test for LGE and MACEs. (D) Egger’s test for ECV and cardiovascular events. (E) Egger’s test for native T1 and MACEs. (DOCX) [file pone.0263378.s003.docx]

**Fig 1S. Egger’s publication bias plots**

A．Egger’s test for LGE and all-cause mortality


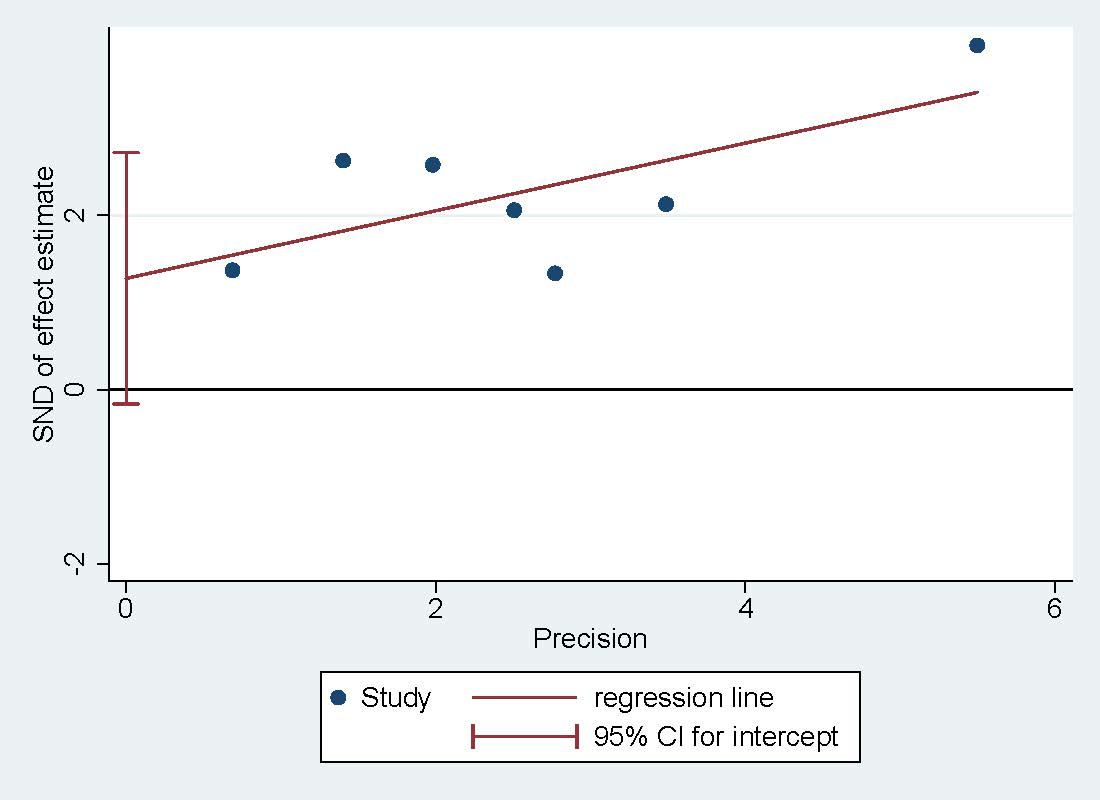


| Number of studies =7 | | | | Root MSE = .7186 | | |
| --- | --- | --- | --- | --- | --- | --- |
| Std_Eff | Coef. | Std.Err | t | P>｜t｜ | [ 95% Conf. Interval ] | |
| slope | 0.3878904 | .187389 | 2.07 | 0.093 | -.0938093 | .86958912 |
| bias | 1.276138 | .5609675 | 2.27 | 0.072 | -.1658752 | .71815 |
| Test of H0: no small-study effects P = 0.072 | | | | | | |

Result:

B．Egger’s test for LGE and cardiac mortality


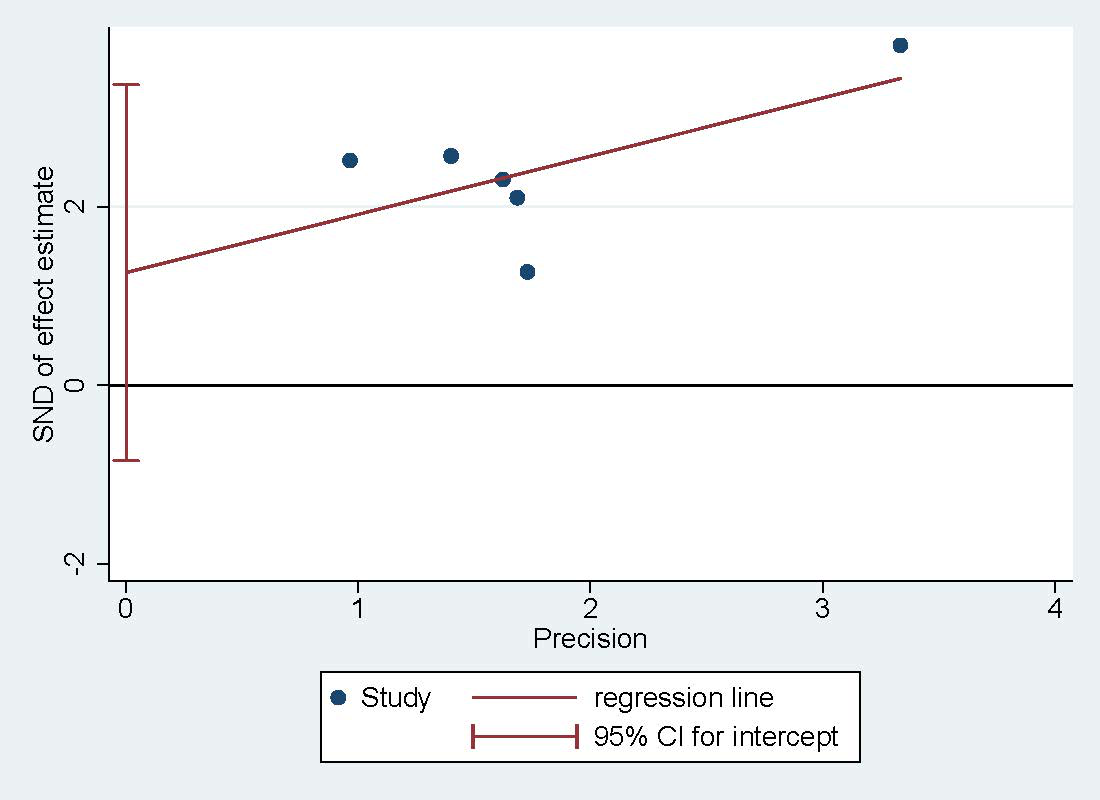


| Number of studies =6 | | | | Root MSE = .7077 | | |
| --- | --- | --- | --- | --- | --- | --- |
| Std_Eff | Coef. | Std.Err | t | P>｜t｜ | [ 95% Conf. Interval ] | |
| slope | 0.6516703 | .3916491 | 1.66 | 0.171 | -.435722 | 1.739063 |
| bias | 1.260187 | .757858 | 1.66 | 0.172 | -.8439642 | 3.364338 |
| Test of H0: no small-study effects P = 0.172 | | | | | | |

Result:

C. Egger’s test for LGE and MACEs


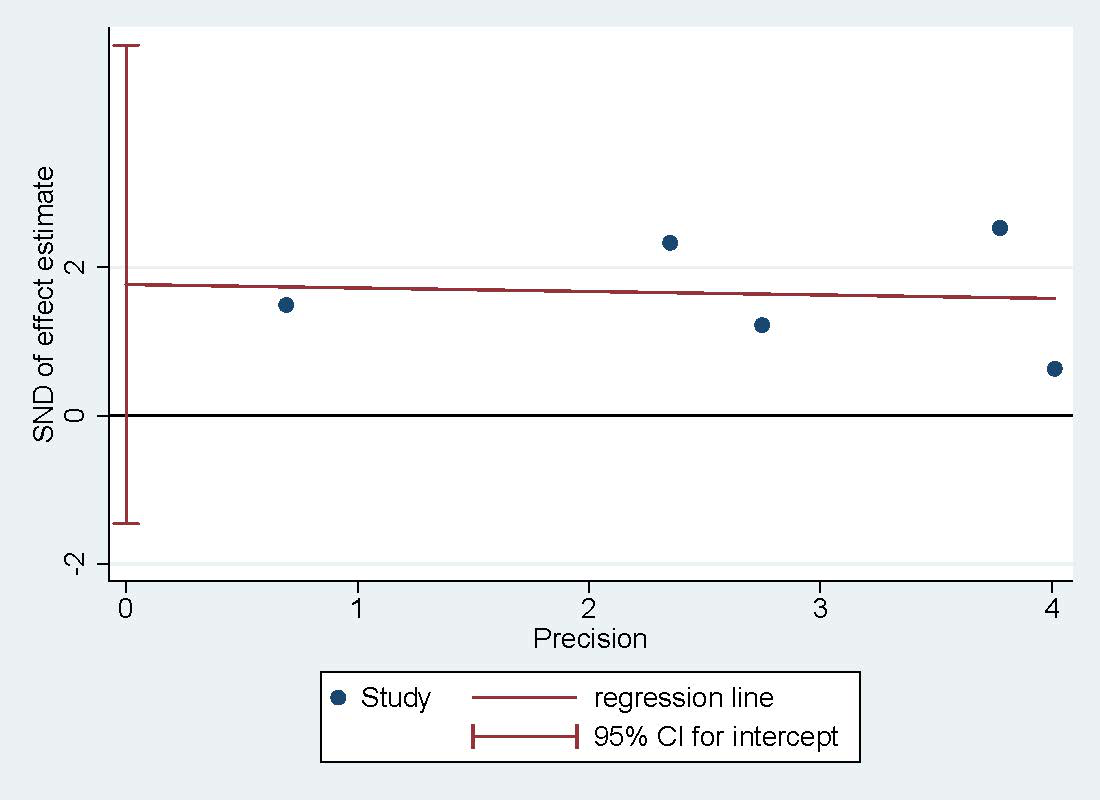


| Number of studies =5 | | | | Root MSE = .9078 | | |
| --- | --- | --- | --- | --- | --- | --- |
| Std_Eff | Coef. | Std.Err | t | P>｜t｜ | [ 95% Conf. Interval ] | |
| slope | -.0469048 | .3421092 | -0.14 | 0.900 | -.1.135649 | 1.041839 |
| bias | 1.770327 | 1.014144 | 1.75 | 0.179 | -1.457133 | 4.997786 |
| Test of H0: no small-study effects P = 0.179 | | | | | | |

Result:

D. Egger’s test for ECV and cardiovascular events


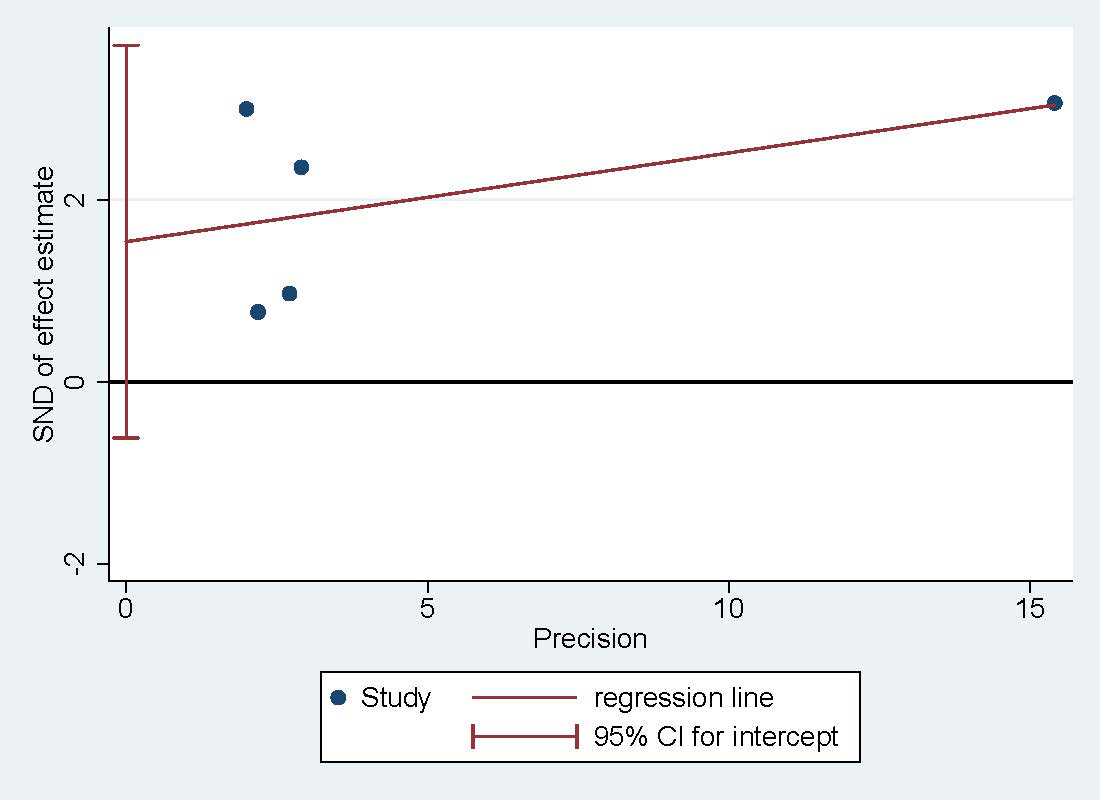


| Number of studies =5 | | | | Root MSE = 1.088 | | |
| --- | --- | --- | --- | --- | --- | --- |
| Std_Eff | Coef. | Std.Err | t | P>｜t｜ | [ 95% Conf. Interval ] | |
| slope | -.0975182 | .093666 | 1.04 | 0.374 | -.2005687 | .3956051 |
| bias | 1.539291 | .6779777 | 2.27 | 0.108 | -.6183365 | 3.696919 |
| Test of H0: no small-study effects P = 0.108 | | | | | | |

Result:

E. Egger’s test for native T1 and MACEs


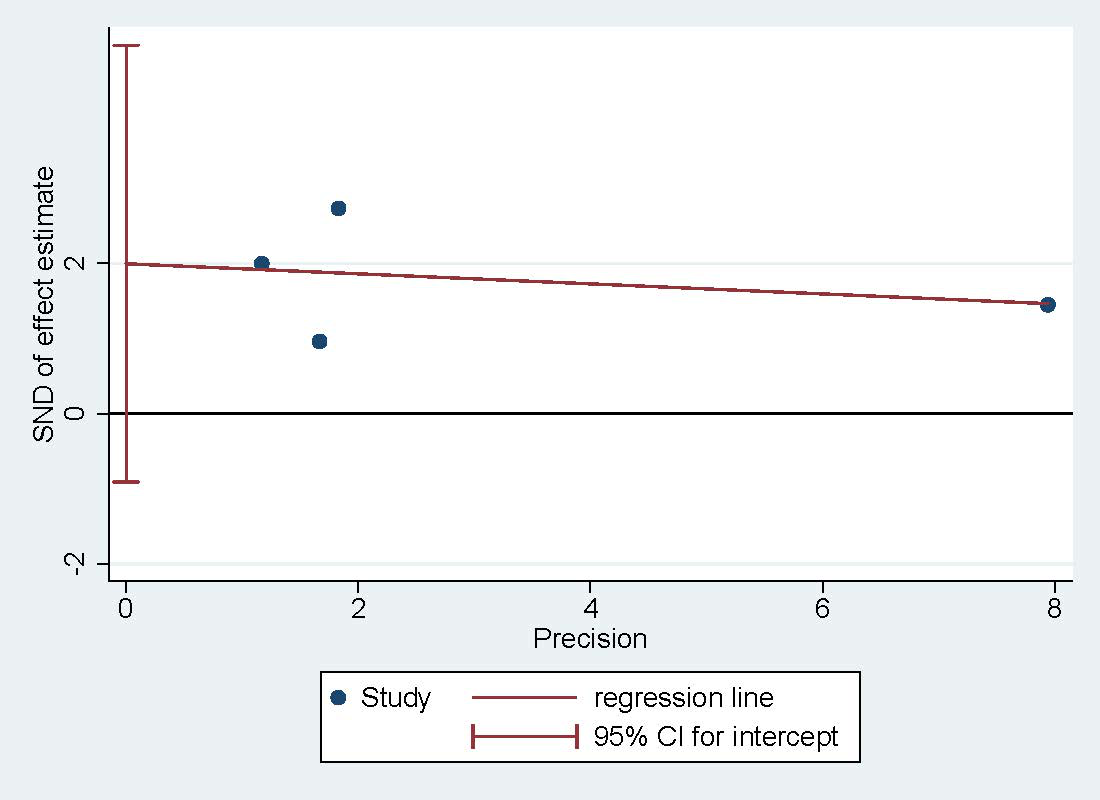


| Number of studies =4 | | | | Root MSE = .8932 | | |
| --- | --- | --- | --- | --- | --- | --- |
| Std_Eff | Coef. | Std.Err | t | P>｜t｜ | [ 95% Conf. Interval ] | |
| slope | -.0668795 | .1608896 | -0.42 | 0.718 | -.7591315 | .6253726 |
| bias | 1.994883 | .6756671 | 2.95 | 0.098 | -.9122778 | 4.902044 |
| Test of H0: no small-study effects P = 0.098 | | | | | | |

Result:
