# Supplementary material for: Prognostic value of cardiac magnetic resonance in patients with aortic stenosis: A systematic review and meta-analysis
Source: PLoS One. 2022 Feb 3;17(2):e0263378. doi: 10.1371/journal.pone.0263378 (PMC8812989; doi:10.1371/journal.pone.0263378)
Supplement: S1 Table — (DOCX) [file pone.0263378.s004.docx]

**SUPPLEMENTARY MATERIAL**

**Table 1S. Risk of bias within studies**

| **NEWCASTLE - OTTAWA QUALITY ASSESSMENT SCALE COHORT STUDIES** | | | | | | | | | | |
| --- | --- | --- | --- | --- | --- | --- | --- | --- | --- | --- |
| **Author** | **year** | **Selection** | | | | **Comparability** | **Outcome** | | | **score** |
|  |  | **Representativeness of the exposed cohort** | **Selection of the nonexposed cohort** | **Ascertainment of exposure** | **Demonstration that outcome of interest was not present at start of study** | **Comparability of cohorts on the**  **basis of the design or analysis** | **Assessment of outcome** | **Follow-up long enough for outcomes to occur** | **Adequacy of follow up of cohorts** |  |
| Hyun-Jung Lee | 2021 | 1 | 1 | 1 | 1 | 1 | 1 | 1 | 1 | 8 |
| Everett | 2020 | 1 | 1 | 1 | 1 | 2 | 1 | 1 | 1 | 9 |
| Hwang | 2019 | 1 | 0 | 1 | 1 | 1 | 1 | 1 | 1 | 7 |
| Agoston-Coldea | 2019 | 1 | 1 | 1 | 1 | 1 | 1 | 1 | 1 | 8 |
| Tarique A. Musa | 2018 | 1 | 1 | 1 | 1 | 2 | 1 | 1 | 1 | 9 |
| Chin | 2017 | 1 | 1 | 1 | 1 | 2 | 1 | 1 | 1 | 9 |
| Lee | 2017 | 1 | 1 | 1 | 1 | 1 | 1 | 1 | 1 | 8 |
| Rajesh | 2017 | 1 | 1 | 1 | 1 | 1 | 0 | 1 | 1 | 7 |
| singh | 2017 | 1 | 1 | 1 | 1 | 1 | 1 | 1 | 1 | 8 |
| Nadjiri | 2016 | 1 | 1 | 1 | 1 | 1 | 1 | 0 | 1 | 7 |
| Barone-Rochette | 2014 | 1 | 1 | 1 | 1 | 2 | 1 | 1 | 1 | 9 |
| Quarto | 2012 | 1 | 1 | 1 | 1 | 1 | 0 | 1 | 1 | 7 |
| Dweck | 2011 | 1 | 1 | 1 | 1 | 1 | 1 | 1 | 1 | 8 |
