# Supplementary material for: Prognostic value of cardiac magnetic resonance in patients with aortic stenosis: A systematic review and meta-analysis
Source: PLoS One. 2022 Feb 3;17(2):e0263378. doi: 10.1371/journal.pone.0263378 (PMC8812989; doi:10.1371/journal.pone.0263378)
Supplement: S2 Table — (DOCX) [file pone.0263378.s005.docx]

**Table 2S. Search strategy (via PubMed)**

| Pubmed | Items |
| --- | --- |
| ((aortic OR aorta) AND stenosis) AND ((magnetic AND resonance AND imaging) OR MRI OR CMR OR MR OR ECV OR (Native T1)) AND (fibrosis OR hypertrophy) AND (death OR survival OR mortality OR onset OR symptoms OR events OR progression OR cardiac OR cardiovascular OR infarct OR decompensation OR edema OR angina) |  |
| Publication year：Before March 31, 2021 | 343 items |
| Humans | 297 items |
| Full text availability | 267 items |
| Adults (>19y) | 193items |
| Article types  - Classical Article  - Clinical Study  - Clinical Trial  - Controlled Clinical Trial  - Multicenter Study  - Observational Study  - Randomized Controlled Trial  - Pragmatic Clinical Trial | 67 items |
| *Filters applied: Full text, Classical Article, Clinical Study, Clinical Trial, Comparative Study, Controlled Clinical Trial, Multicenter Study, Observational Study, Randomized Controlled Trial, Humans, Adult: 19+ years, from 1000/1/1 - 2021/3/31.* |  |
